# Histone acetyltransferase and DNA methyltransferase expression in response to LPS stimulation

**DOI:** 10.1186/cc12988

**Published:** 2013-11-05

**Authors:** Ester Correia Sarmento Rios, Francisco Garcia Soriano

**Affiliations:** 1 Departamento de Clínica Médica da Universidade de São Paulo, SP, Brazil

## Background

Tolerance is a defense strategy capable of reducing the proinflammatory impact of infection. Tolerance capacity differs among the different tissues. It is known that epigenetic regulation is cell type specific. The cell machinery regulates the expression and activity of the enzymes that regulate chromatin openness. Understanding the epigenetic mechanism activated by different doses of LPS is important to define new approaches for the treatment of systemic infections. The objective of this work was to study the LPS-induced epigenetic response, analyzing the expression of histone acetyltransferases (HAT) and DNA methyltransferases (DNMTs).

## Materials and methods

THP-1 human promonocytes were cultivated in RPMI (C group), submitted to different doses of LPS (T group - tolerance with 500 ng/ml during 24 hours and challenge with 1 µg/ml during 24 hours; D group - 1 µg/ml during 24 hours). The inhibition of nitric oxide production was performed with LNAME (100 µM). Male Balb C mice (8 weeks old) were divided into two groups: C group - without manipulation; D group - received 5 mg/kg LPS. The spleens were collected 48 hours after. The HAT, DNMTs, lysine acetylated and histone H3 acetylated amounts were determinate by western blot. The results represent three similar experiments. The statistical analysis was performed by ANOVA. Research protocol number 0950/09 was approved by the ethics committee.

## Results

The challenge with LPS reduced the expression of DNMT1 in THP1 cells. However, the tolerance increased the amount of this enzyme (25%). Challenge with LPS reduces DNTM3a production (50%) in mice spleen. The expression of HAT was reduced (50%) in the T group and this event was NO dependent. The LPS addition to THP1 culture decreases the production of acetylated lysine (*P *< 0.05) in a dose-dependent way (68% and 33% with 0, 1 and 5 µg/ml LPS respectively). Low doses of LPS reduce the acetylation of histone H3 (30% and 60% with 500 ng and 1 µg/ml LPS respectively).

## Conclusions

Different concentrations of LPS are required for selective regulation of subsequent LPS-stimulated epigenetic mechanisms.

